# Arginine and Lysine Transporters Are Essential for *Trypanosoma brucei*

**DOI:** 10.1371/journal.pone.0168775

**Published:** 2017-01-03

**Authors:** Christoph Mathieu, Juan P. Macêdo, Daniel Hürlimann, Corina Wirdnam, Alexander C. Haindrich, Marianne Suter Grotemeyer, Amaia González-Salgado, Remo S. Schmidt, Ehud Inbar, Pascal Mäser, Peter Bütikofer, Dan Zilberstein, Doris Rentsch

**Affiliations:** 1 Institute of Plant Sciences, University of Bern, Bern, Switzerland; 2 Institute of Biochemistry and Molecular Medicine, University of Bern, Bern, Switzerland; 3 Swiss Tropical and Public Health Institute and University of Basel, Basel, Switzerland; 4 Faculty of Biology, Technion-Israel Institute of Technology, Haifa, Israel; Instituto Butantan, BRAZIL

## Abstract

For *Trypanosoma brucei* arginine and lysine are essential amino acids and therefore have to be imported from the host. Heterologous expression in *Saccharomyces cerevisiae* mutants identified cationic amino acid transporters among members of the *T*. *brucei* AAAP (amino acid/auxin permease) family. TbAAT5-3 showed high affinity arginine uptake (*K*_m_ 3.6 ± 0.4 μM) and high selectivity for L-arginine. L-arginine transport was reduced by a 10-times excess of L-arginine, homo-arginine, canavanine or arginine-β-naphthylamide, while lysine was inhibitory only at 100-times excess, and histidine or ornithine did not reduce arginine uptake rates significantly. TbAAT16-1 is a high affinity (*K*_m_ 4.3 ± 0.5 μM) and highly selective L-lysine transporter and of the compounds tested, only L-lysine and thialysine were competing for L-lysine uptake. TbAAT5-3 and TbAAT16-1 are expressed in both procyclic and bloodstream form *T*. *brucei* and cMyc-tagged proteins indicate localization at the plasma membrane. RNAi-mediated down-regulation of *TbAAT5* and *TbAAT16* in bloodstream form trypanosomes resulted in growth arrest, demonstrating that TbAAT5-mediated arginine and TbAAT16-mediated lysine transport are essential for *T*. *brucei*. Growth of induced RNAi lines could partially be rescued by supplementing a surplus of arginine or lysine, respectively, while addition of both amino acids was less efficient. Single and double RNAi lines indicate that additional low affinity uptake systems for arginine and lysine are present in *T*. *brucei*.

## Introduction

Trypanosomes are unicellular, flagellated eukaryotic parasites that cycle between different hosts and cause severe diseases. *Trypanosoma brucei* subspecies cause human African trypanosomiasis and also affect wild game and, more severely, cattle [1]. Recent estimates from the Food and Agriculture Organization on economic losses of agricultural gross domestic product due to animal trypanosomiasis reaches US$ 4.75 billion per year [2]. *T*. *brucei* are transmitted to mammalian hosts through the bite of an infected tsetse fly. The environments change dramatically upon transfer from the insect to the mammalian host or *vice versa*, and trypanosomes must adapt nutrient uptake accordingly. In the insect host, glucose is limited and therefore the trypanosomes rely on amino acids as energy source, whereas in the vertebrate host, trypanosomes use glucose as energy source and oxidative phosphorylation is absent [3,4]. Amino acid uptake is of fundamental importance for trypanosomes as they are auxotroph for a number of amino acids, including the proteinogenic amino acids arginine and lysine [5].

Increased transport activity for arginine upon amino acid starvation was found in parasites and in mammalian cells. *Leishmania* arginine transporter LdAAP3 mRNA and protein levels were elevated in promastigotes when starved for arginine. This process looks similar to mammalian cationic amino acid transporter 1 (CAT-1) regulation upon deprivation of amino acids [6]. It has been suggested that LdAAP3 contributes to parasite virulence by increased arginine uptake [6]. The arginine-deprivation response resulting in LdAAP3 up-regulation was recently shown to be mediated through a MAPK2-dependent signaling pathway [7]. While arginine is important for polyamine biosynthesis in *Leishmania*, the absence of a functional arginase homolog and the essentiality of ornithine decarboxylase (ODC) in *T*. *brucei* indicate that the latter rely on ornithine as precursor for polyamine biosynthesis [8–11], though in *T*. *brucei* ornithine synthesis from arginine seems possible when ornithine is limiting [12]. Furthermore in *T*. *cruzi* and *T*. *brucei*, arginine kinase converts arginine in an ATP-dependent reaction to phosphoarginine, contributing to the energy buffering of cells [13,14].

A variety of 48 putative amino acid transporter genes (including three pseudogenes) belonging to two different families are predicted in the genome of *T*. *brucei* (TriTrypDB [15–17]). Most genes encoding putative amino acid transporters belong to the amino acid/auxin permease (AAAP, TC 2.A.18) family and are clustered in 17 loci distributed over the 11 chromosomes [17]. Additionally, a member of the APC (amino acid-polyamine-organocation, TC 2.A.3) family was identified in the *T*. *brucei* genome [16]. So far, only one *T*. *brucei* amino acid transporter (i.e. TbAAT6) has been functionally characterized and linked to low affinity, broad specificity amino acid and eflornithine uptake, and consequently, eflornithine resistance [18–21].

Arginine and lysine transporters belonging to the AAAP family have been characterized in *Leishmania* and *T*. *cruzi* [6,22–25]. Interestingly, most of the functionally characterized *L*. *donovani* and *T*. *cruzi* arginine and lysine transporters are highly selective for their substrates. Expression of *Leishmania* LdAAP3 and *T*. *cruzi* TcAAAP411 in *Saccharomyces cerevisiae* mutants showed high selectivity and high affinity for arginine, i.e. *K*_m_ of 1.9 μM and 32 μM, respectively [22,23], and the affinity of LdAAP7 for lysine was 7.4 μM [24]. Recently the *K*_m_ of *T*. *cruzi* CAT1.1 for arginine was shown to be 85 μM, while the same transporter showed lower affinity for ornithine (*K*_m_ 1.7 mM) [25].

In contrast to the transporters for lysine or arginine characterized from parasites, cationic amino acids are transported by the same transport system in mammalian cells, i.e. members of SLC7 family, also referred to as the APC (TC 2.A.3) transporter family [26]. While rBAT, a member of the L-type amino acid transporter (LAT) subgroup, mediates exchange of cationic amino acids and cysteine against neutral amino acids, mammalian CAT1 to 3, which are members of the cationic amino acid transporters (CAT) subgroup, mediate facilitated uptake of cationic amino acids and are trans-stimulated to various degrees [26]. Apparent affinities of CAT members for arginine, lysine and ornithine are comparable and in the high affinity (40–450 μM; CAT1, CAT2B, CAT3) or low affinity (2–5 mM; CAT2A) range [27]. Only histidine is recognized differently by these transporters.

In the present work we report the identification and functional characterization of high affinity arginine and lysine permeases from *T*. *brucei* and show that in contrast to the mammalian transporters for cationic amino acids, the *T*. *brucei* arginine and lysine transporters are highly selective. Furthermore, we show that uptake of both, arginine and lysine, is essential for parasite survival in culture.

## Materials and Methods

Animal experiments (in Prof. E. Sigel's lab) were carried out in strict accordance to the Swiss ethical guidelines, and have been approved by the local committee of the Canton Bern Kantonstierarzt, Kantonaler Veterinärdienst Bern (BE85/15). Surgery of female adult *Xenopus laevis* was done under anesthesia (0.2% tricaine solution). Oocytes were prepared, injected and defolliculated as described previously [28].

### Phylogenetic analysis

Annotated amino acid transporters (after [17]) in the genome of *T*. *brucei* TREU 927 within TriTrypDB were screened for Pfam families. Most amino acid transporters were part of Pfam family PF01490, Aa_trans (Transmembrane amino acid transporter protein). This family was used to screen the best-curated genomes in trypanosomatids, namely *Trypanosoma cruzi* CL Brener non-Esmeraldo-like, *T*. *cruzi* CL Brener Esmeraldo-like, *Leishmania major* strain Friedlin and *T*. *brucei* TREU 927, for genes that are members of this family. Using the coding sequences of the resulting 109 genes, we generated a codon-based alignment using MUSCLE [29]. Based on this analysis, we grew a phylogenetic tree by the Neighbor-Joining method. Both of these steps were carried out using the MEGA6 software [30]. Based on this tree, a limited tree showing representative genes was generated. The representative genes include: genes studied here, their homologs in the aforementioned genomes, characterized homologs from *T*. *cruzi*, *L*. *donovani* as well as TbAAT6 [21–25]. The amino acid sequences were used to calculate pairwise global alignment scores using the Needleman-Wunsch algorithm utilizing the BLOSUM62 matrix, a gap-opening penalty of 10 and a gap extension penalty of 0.5.

### *Saccharomyces cerevisiae* transformation

Transformation of *S*. *cerevisiae* was performed according to [31]. *S*. *cerevisiae* mutants: JT16 (*Mat*a, *hip1-614*, *his4-401*, *can1*, *ino1*, *ura3-*52; [32]), 22Δ6AAL (*Mat*α, *ura3-1*, *gap1-1*, *put4-1*, *uga4-1*, *can1::hisG*, *lyp1/alp1::hisG*, *lys2::hisG*; [33]), 21.983c (*MAT*a, *gap1-1*, *can1-1*, *ura3*; Hein and André, 1997). 22574d (*Mat*α, *ura3-1*, *gap1-1*, *put4-1*, *uga4-1*; [34]), 30.537a (*Mat*α, *gap1-1*, *dip5::kanMX2*, *ura3*; supplied by Professor Bruno André, Université Libre de Bruxelles), YDR544 (*Mat*α, *ura3-1*, *gap1-1*, *put4-1*, *uga4-1*, *ssy1::kanMX*; [22]), Y01543 (*Mat*a, *his3Δ1*, *leu2Δ0*, *met15Δ0*, *ura3Δ0*, *YLL055w::kanMX4;* EUROSCARF). Media for selective and non-selective growth were as described previously [21]. For transport experiments strain 22Δ7AA (*Mat*α, *ura3-1*, *gap1-1*, *put4-1*, *uga4-1*, *can1::hisG*, *lyp1/alp1::hisG*, *hip1::hisG*; [33]) was grown in minimal medium [MM: 1.7 g L^-1^ yeast nitrogen base without amino acids and without ammonium sulfate (Difco), and 20 g L^-1^ glucose] containing 1 g L^-1^ urea.

### DNA and RNA-work

The 1377 bp TbAAT5-ORFs were amplified by PCR using genomic DNA of *T*. *b*. *brucei* Lister strain 427, PfuUltra DNA polymerase (Stratagene), and primers 5’-CGGAATTCATGCTGAGCCCTACAGAACCATTAG-3’, 5’-CGCGGATCCTTACACAGCCACATTGTAGATTGAGG-3’. The fragments were cleaved with *Eco*RI and *Bam*HI and cloned into the respective sites of the *S*. *cerevisiae* expression vector pDR197 [35]. For tetracycline-inducible RNA-interference (RNAi) against *TbAAT5* in procyclic forms, a 596 bp fragment of the ORF Tb427.08.4720 (nucleotide position 234 to 829) was amplified using primers 5'- GGCCAAGCTTGGATCCCATAAGTCTCGCTATCGCTTTTCAG, 5'- GGCCTCTAGACTCGAGGTTTGCGCATCTCCGAGTAAATGG, and cloned into the stem-loop RNAi vector pALC14 (a derivative of pLEW100 [36] containing puromycin as selectable marker). In bloodstream forms (BSF) the *TbAAT5*-RNAi cassette mentioned above was similarly cloned into the stem-loop RNAi vector pMS14v5 [37] (a derivative of pLEW100 [36] containing phleomycin as selectable marker). All RNAi vectors were linearized with *Not*I prior to parasite transfection. For expression in oocytes, the *TbAAT5* ORF was cloned into vector pBF1 [38]. Therefore Tb427.08.4720 was excised by *Eco*RI/*Bam*HI from pDR-Tb427.08.4720 and cloned into *Eco*RI/*Bgl*II of pBF1. pBF1-Tb427.08.4720 was linearized with *Mlu*I and cRNA synthesized by using the Sp6 mMessage mMachine kit (Ambion, Austin, TX). RNA was purified by LiCl precipitation and stored at -80°C.

The 1389 bp TbAAT16-ORFs were amplified by PCR using genomic DNA of *T*. *brucei brucei* Lister strain 427, and primers 5’-CGGAATTCATGTCACCCAGGGCAACAGAGCC-3’ and 5’-CGCGGATCCCTACTTCTTAACCTCGTCGTAAATGGTTAATGCC-3’. The fragments were cleaved with *Eco*RI and *Bam*HI and cloned into the respective sites of the *S*. *cerevisiae* expression vector pDR197. Tb427.tmp.01.7520 was not amplified using the primers above. Instead, we amplified Tb427.tmp.01.7520_G_343_V containing a single amino acid difference, which was mutagenized to obtain Tb427tmp.01.7520. Therefore T at position 1029 was mutated to G with the QuickChangeRII Site-Directed Mutagenesis Kit (Stratagene, La Jolla, USA) using primers 5’-CGCTATCAAGATATGCG**G**AGGATTCGCTATTTGCATCC-3’ and 5’-GCGATAGTTCTATACGC**C**TCCTAAGCGATAAACGTAGG-3’. For RNAi a 600 bp fragment of Tb427tmp01.7500 (ORF position 323 to 922) was amplified using primers: 5'-GGCCAAGCTTGGATCCTGTACTCTATCCGCATACTCGTG-3’ and 5'-GGCCTCTAGACTCGAGGATAACCGAAGATACCAGACAAGAAATAC-3’ and cloned into pMS14v5 [37]. For cRNA synthesis and subsequent expression in oocytes Tb427tmp.01.7500 was excised from pDR197 with *Eco*RI and *Bam*HI and inserted into the corresponding site of the vector pBF1 [38]. All constructs were verified by sequencing.

For tightly-regulated inducible expression of cMyc-TbAAT5 and cMyc-TbAAT16, the ORFs were cloned into a modified pLEW100 vector [36], in which the phleomycin resistance gene has been replaced by the puromycin resistance gene and the triple cMyc cassette [39] was inserted to allow N-terminal tagging [40].

### Transport assays

Transport assays in *S*. *cerevisiae* strain 21.983c and 22Δ7AA were performed as described previously [41] with slight modifications. Cells were grown to a density of OD_578_ up to 0.6, washed twice with water and resuspended in buffer A (1/10 initial volume; 0.6 M sorbitol and 50 mM potassium phosphate, different pH values). Prior to the transport assay, cells were preincubated at 30°C for 6 min in the presence of 100 mM glucose. To start the transport assay, cells (100 to 130 μl) were added to an equal volume of buffer with different concentrations of L-arginine or L-lysine and 3.7–18.5 kBq L-[^3^H]-arginine (1.48–2.22 TBq mmol^-1^) or 37–74 kBq L-[^3^H]-lysine (2.2–3.2 TBq mmol^-1^) per assay. For some experiments, competitors as specified in the results section were added.

Samples (48 μl) were transferred after 30 sec, 1 min, 2 min, 4 min (and 6 min) to 4 ml ice-cold buffer A, filtrated on glass fiber filters and washed twice with 4 ml ice-cold buffer A. The uptake of tritium-labeled substrates was determined by liquid scintillation spectrometry. In all experiments, uptake was linear over the time period investigated and transport rates were calculated from at least four different time-points. Uptake rates of *S*. *cerevisiae* transformed with ‘empty vector’ was subtracted as background. Kinetic parameters were calculated using the Michaelis-Menten equation V = *V*_max_ x [S] x (*K*_m_ + [S])^-1^.

### Expression in *Xenopus laevis* oocytes

Preparation of the *X*. *laevis* oocytes, cRNA synthesis and injection, setup and two-electrode voltage clamping were essentially as described previously [21,28]. 50 nl of pBF1-Tb427.08.4720 (TbAAT5-3) or pBF1- Tb427tmp.01.7500 (TbAAT16-1) cRNA (50 ng) was injected per oocyte. Oocytes were clamped at a membrane potential of -80 mV. For Tb427.08.4720, currents were normalized to currents induced by 50 μM arginine (pH 7.4, *V*_m_ -80 mV) in Na^+^-Ringer buffer (115 mM NaCl, 2 mM KCl, 1.8 mM CaCl_2_, 1 mM MgCl_2_ and 5 mM HEPES) to correct for different expression levels between oocytes. Calculations and curve fittings were as described [28]. Experiments to test for co-transport of Na^+^ and K^+^ were performed in buffer containing 1.8 mM CaCl_2_, 1 mM MgCl_2_, 5 mM HEPES and 117 mM choline chloride (choline Ringer). Substrates were added to the buffer solutions as indicated, and the necessary pH adjustments were made with tris(hydroxymethyl)aminomethane (TRIS). Values represent mean ± SE of at least 5 oocytes from three batches of oocytes.

### *T*. *b*. *brucei* culture

*T*. *brucei* bloodstream form (BSF) New York single marker (NY-SM, [36]) were cultured at 37°C and 5% CO_2_ in HMI-9 medium supplemented with 10% (v/v) of heat-inactivated FBS (Gibco, Basel, Switzerland). NY-SM was the parent line to obtain the RNAi cell lines. *TbAAT5*-RNAi and *TbAAT16-*RNAi BSF lines were selected and cultured in the presence of 1 μg ml^-1^ geneticin (G418) and 1.5 μg ml^-1^ phleomycin. TbAAT5/16 dRNAi clones were selected and cultured in the presence of 1 μg ml^-1^ geneticin (G418), 1.5 μg ml^-1^ phleomycin and 0.1 μg ml^-1^ puromycin.

*T*. *brucei* 29–13 procyclic forms (PCF) [36] were cultured at 27°C in SDM-79 supplemented with 10% (v/v) heat-inactivated FBS (Gibco), in the presence of 25 μg ml^-1^ hygromycin and 15 μg ml^-1^ G418. In this study 29–13 cells were the parental cell line used to obtain RNAi and over-expressing PCF *T*. *brucei*. *TbAAT5*-RNAi PCF and cMyc-TbAAT5 or cMyc-TbAAT16 over-expressing PCFs were selected and cultured with 1 μg ml^-1^ puromycin.

### *T*. *b*. *brucei* stable transfection

For transfection, NY-SM BSF *T*. *brucei* were harvested at mid-log phase and washed once in phosphate-buffered saline (PBS; 137 mM NaCl, 2.7 mM KCl, 10 mM Na_2_HPO_4_, 1.76 mM KH_2_PO_4_, pH 7.2). Parasites were resuspended in 100 μl electroporation buffer (90 mM Na_2_HPO_4_, 5 mM KCl, 0.15 mM CaCl_2_, 50 mM HEPES, pH 7.3) containing 7–10 μg of DNA. Electroporation (pulse code FI-115, “Primary Cell P3” solution) was performed in 100 μl nucleocuvettes using Lonza 4D Nucleofector System. Cells were immediately inoculated in 10 ml culture medium, diluted 1:200–400 and plated in 24-well plates (1 mL per well). After 24 h, the corresponding antibiotics were added, i.e. 2.5 μg ml^-1^ phleomycin for selection of *TbAAT16* RNAi cell lines, or 0.1 μg ml^-1^ puromycin for selection of *TbAAT5* RNAi cell line. Double-RNAi cell line was selected in presence of both antibiotics.

### Quantitative RT-PCR

Total RNA was isolated using the SV RNA isolation system (Promega, Madison, USA) following the manufacturer instructions. RNA samples were treated with DNase I (Roche, Basel, Switzerland) for 15 min at 37°C, followed by phenol/chloroform extraction and ethanol precipitation. Absence of gDNA contamination was confirmed by PCR. DNase I-treated RNA (0.5 μg) was used for cDNA synthesis using Takara PrimeScript reverse transcriptase (Takara, Shiga, Japan). Quantitative PCR was performed using a LightCycler 480 System (Roche). The reaction mixtures consisted of 1x Sybr green premix, *Ex taq* (RR420L, Takara) and 0.2 μM forward and reverse primers. *TbAAT5* primers used for qRT-PCR analysis of RNAi lines, AAT5F 5’-ATCATTGCGGGTTTCTTCGG-3’ and AAT5R 5’- ACGCAATTGCCATCATCACG-3’. Primers used to compare stage-specific expression of *TbAAT5* members: 4700F 5’-GGCGTGTCCAGTAAATTTCCAC-3’ and 4700R 5’-AGACCAACTGCTGTCTTCTCTG-3’; 4710-40F 5’-TTCCCACAGGGTTAAGTGAAGG-3’ and 4710-40R 5’-GCTGGAGTAACGTATTGTTGGC-3’. *TbAAT16* primers used, AAT16F 5’-GGAATGATCTAGAAGGCTCCGTAC-3’ and AAT16R: 5’- CTTGATAGCGATGCCAACATACCC-3’. Real-time PCR analyses were performed in duplicates of three different cDNA dilutions and PCR efficiencies were carefully controlled (>90%). Telomerase reverse transcriptase (TERT, Tb927.11.10190) was used as reference using primers described before [42]. For comparisons of the expression levels in BSF and PCF, a second control gene (C1, Tb927.10.12970), described to be stably expressed in different life-cycle stages of *T*. *brucei* [43] was used in addition to TERT.

### Immunolocalization

For immunolocalization of N-terminally cMyc-tagged TbAAT5-3 and TbAAT16-1, PCF over-expressing TbAAT5-3 (Tb427.08.4720) or TbAAT16-1 (Tb427tmp.01.7500) were fixed on poly-lysine slides with 4% (w/v) paraformaldehyde, followed by permeabilization with 0.2% (v/v) TX-100 in PBS. Antibody against cMyc (monoclonal antibody, mouse, clone 9E10, Santa Cruz Biotechnology) was applied at a dilution of 1:1000 in 5% (w/v) skim milk powder in PBS, followed by Alexa Fluor^®^ 488, Goat anti-Mouse IgG (H+L) (Life Technologies), at a dilution of 1:1000. Coverslips were mounted with Vectashield^®^ containing 4’,6-diamidino-2’-phenylindole (DAPI; Vector Laboratories) and images were obtained using a confocal microscope (Leica DM RXE, equipped with a Leica TCS SP2 confocal scanner) in sequential scanning mode. Alexa Fluor^®^ 488 was excited with a wavelength of 488 nm and emission was detected at 500–600 nm. DAPI was detected at 410–610 nm after excitation at a wavelength of 405 nm. Final images were analyzed using Fiji software.

## Results

### *T*. *brucei* TbAAT5 and TbAAT16 are homologs of arginine and lysine transporters in other trypanosomatids

**Fig 1:**
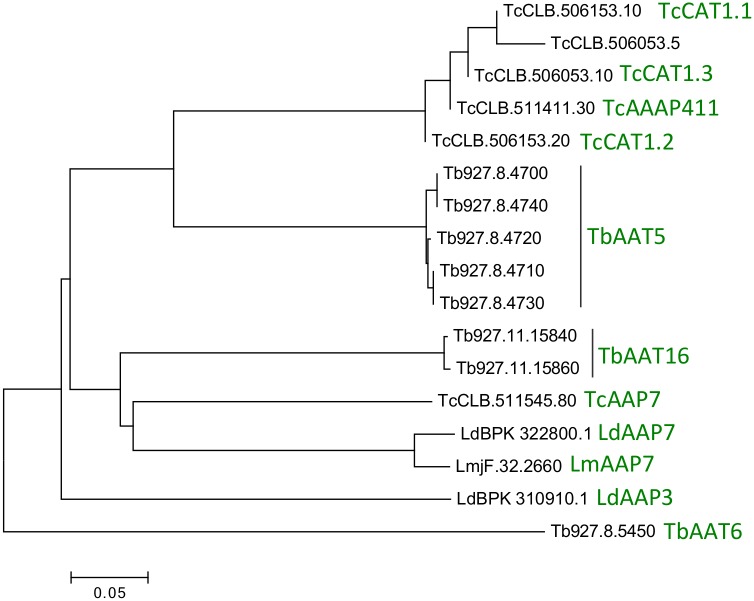
Phenogram of *T*. *brucei* amino acid transporters. Phylogenetic relationship of members of the *T*. *brucei* TREU 927 AAT5 (Tb927.8.4700-4740) and AAT16 (Tb927.11.15840, Tb927.11.15860) families and characterized arginine and lysine transporters from *T*. *cruzi* and *L*. *donovani*: TcAAAP411, (TcCLB.511411.30 [23]), TcAAP7 (TcCLB.511545.80 [24]), TcCAT1.1, TcCAT1.2, TcCAT1.3 (TcCLB.506153.10, TcCLB.506153.20, TcCLB.506053.10 [25]); LdAAP7 (LdBPK_322800.1 [24]) and LdAAP3 (LdBPK_310910.1 [22]). Further shown are AAP7 in *L*. *major*, LmAAP7 (LmjF.32.2660); the characterized, but more distantly related transporter of eflornithine and neutral amino acids, TbAAT6 (Tb927.8.5450, [18–21]); an annotated fragment with high similarity to TcCAT1.1 (TcCLB.506053.5). Scale bar represents substitutions per site.

The *TbAAT5* and *TbAAT16* loci are synthenic in the reference *T*. *brucei* strain TREU927 and in Lister strain 427 used in our experiments. The *TbAAT5* locus of *T*. *brucei* is located on chromosome 8 and consists of 5 full-length gene members and one pseudogene [17], i.e. Tb427.08.4700, Tb427.08.4710, Tb427.08.4720, Tb427.08.4730, Tb427.08.4740, and the 120 nt long pseudogene Tb427.08.4750 (S1 Fig). It should be noted that the full-length genes mentioned above are annotated differently in TriTrypDB for *T*. *brucei* strains 927 and 427. RNA-seq data [44–46] support ORFs of 1377 nt for all full-length *TbAAT5* members, as shown in the TREU927 annotation. Our nucleotide numbering of *TbAAT5* members in strain 427 refers to the TREU927 annotation (S1 Table, S1 Fig). The amino acid sequences of Tb427.08.4710 and Tb427.08.4720 are identical (not considering the unresolved 6 nucleotides; http://tritrypdb.org), while in Tb427.08.4730 a proline at position 43 is replaced by leucine (S1 Table). Tb427.08.4700 is identical to Tb427.08.4740 except for a predicted deletion of 4 nucleotides (at positions 60–63; S1 Table), which is not present in Tb927.8.4700 and would generate a truncated transporter in strain 427. The most divergent members, i.e. Tb427.08.4730 and Tb427.08.4740, differ from each other in at least 7 amino acids (S1 Table). Phylogenetic analyses revealed that the TbAAT5 proteins are homologs of the functionally characterized *T*. *cruzi* arginine transporter TcAAAP411 (TcCLB.511411.30 [23]; 76–78% similarity) and TcCAT1.1 to TcCAT1.3 [25] (70–76% similarity), but show a lower degree of similarity to the *Leishmania* arginine transporter LdAAP3 (LdBPK_310910.1 [6,22]; 60–62% similarity) (Fig 1 and S2 Table).

The *TbAAT16* locus is located on chromosome 11 and contains two genes, Tb427tmp.01.7500 and Tb427tmp.01.7520 (Tb927.11.15840 and Tb927.11.15860 are the syntenic genes in *T*. *brucei* TREU927) [17] (S1 Fig). TbAAT16 gene products differ from each other by only 3 amino acids (S3 Table). TbAAT16 shares high similarity to the characterized lysine transporters in *T*. *cruzi* and *Leishmania* (TcAAP7 and LdAAP7, respectively; 68–70% similarity (S2 Table), [24]). On the other hand the degree of similarity of TbAAT16 is slightly higher to the above-mentioned arginine transporters from *T*. *brucei* (TbAAT5; 62–63% similarity) and *T*. *cruzi* (TcAAAP411, TcCAT1.1; 62% similarity) than to the *L*. *donovani* arginine transporter LdAAP3 (59% similarity; Fig 1 and S2 Table).

PCR using genomic DNA from *T*. *brucei* strain 427 amplified three different ORFs for *TbAAT5*, i.e. (1) Tb427.08.4720, the unresolved nucleotide at position 96 was identified as cytosine, resulting in aspartate (S1 Table); (2) Tb427.08.4720_D20N in which a single guanine was replaced by an adenine, changing the aspartate at position 20 of Tb427.08.4720 into an asparagine; and (3) Tb427.08.4740, the unresolved nucleotide at position 96 was identified as guanine, resulting in glutamate (S1 Table).

For TbAAT16, three variants were found; the first amino acid sequence corresponded to the database annotation of Tb427tmp.01.7500; the second Tb427tmp.01.7500_V343G, differed from Tb427tmp.01.7500 in seven nucleotides, but only one (nt 1028) resulted in a single amino acid difference, i.e. a valine at position 343 was replaced by glycine; the third, Tb427tmp.01.7520_G343V, differed from the database annotation of Tb427tmp.01.7520 in one amino acid. Since Tb427tmp.01.7520 was not amplified in our analysis, it was generated by site directed mutagenesis of Tb427tmp.01.7520_G343V (S3 Table).

Comparable to predictions for other members of the AAAP family [47], membrane topology prediction platforms (Phobius [48] and HMMTOP [49]) suggested 11 transmembrane domains for the full-length TbAAT5 proteins and 10 or 11 transmembrane domains for TbAAT16 proteins.

### TbAAT5 and TbAAT16 expressed in *S*. *cerevisiae* mediate uptake of cationic amino acids

**Fig 2:**
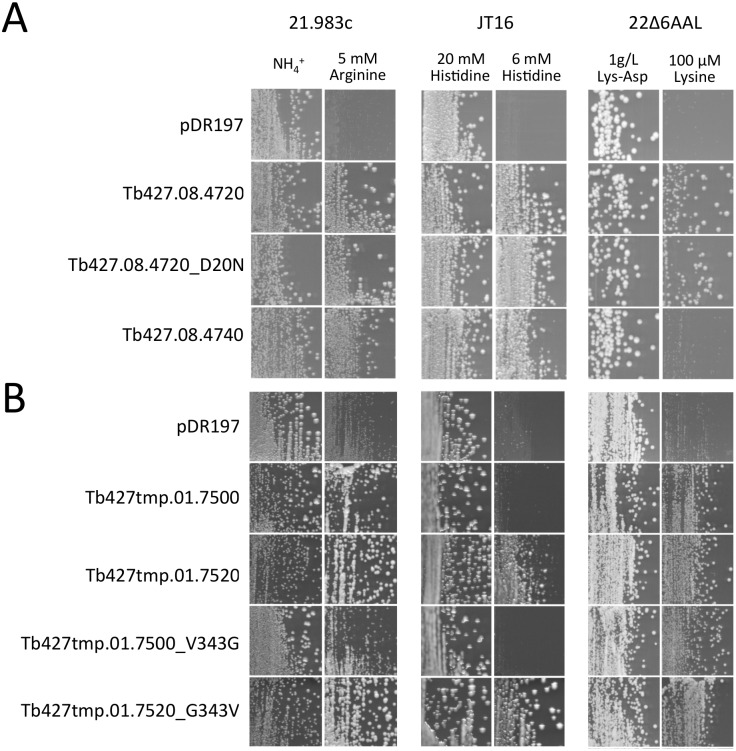
TbAAT5 and TbAAT16 mediate growth of *S*. *cerevisiae* mutants on arginine, lysine and/or histidine. Growth of *S*. *cerevisiae* mutants transformed with the vector pDR197 or pDR197 harboring different members of the *T*. *brucei* (A) AAT5 and (B) AAT16 family. *S*. *cerevisiae* was grown for 3 days on non-selective (left) and selective (right) medium. Strains allow selection for arginine (21.983c), histidine (JT16) and lysine (22Δ6AAL) transport, respectively. Lysine supplemented as lys-asp dipeptide was used as source of lysine in the non-selective medium for strain 22Δ6AAL.

To identify potential substrates of TbAAT5 and TbAAT16, the isolated ORFs were expressed in *S*. *cerevisiae* mutants impaired in the uptake of different amino acids. Interestingly, all three *TbAAT5* gene products (Tb427.08.4720, Tb427.08.4720_D20N and Tb427.08.4740) mediated growth on arginine and histidine, but only Tb427.08.4720 and Tb427.08.4720_D20N mediated growth on lysine (Fig 2A).

The *TbAAT16* gene products Tb427tmp.01.7500, Tb427tmp.01.7500_V343G, Tb427tmp.01.7520_G343V and Tb427tmp.01.7520 mediated growth on lysine and arginine, while Tb427tmp.01.7520_G343V and Tb427tmp.01.7520 complemented histidine transport deficiency (Fig 2B).

None of the ORFs mediated growth on any of the other tested amino acids (i.e. proline, alanine, cysteine, γ-aminobutyric acid, glutamate, aspartate, methionine, phenylalanine, valine, isoleucine, leucine, tryptophan, tyrosine, threonine and citrulline, data not shown). These results indicated that TbAAT5 and TbAAT16 are selective cationic amino acid transporters.

As it is unclear whether the different single nucleotide polymorphisms represent strain differences or may originate from (fusion) PCR events, for further functional analysis we decided to focus on one representative each that corresponded to the database amino acid sequence, i.e. Tb427.08.4720 (TbAAT5-3) and Tb427tmp.01.7500 (TbAAT16-1).

**Fig 3:**
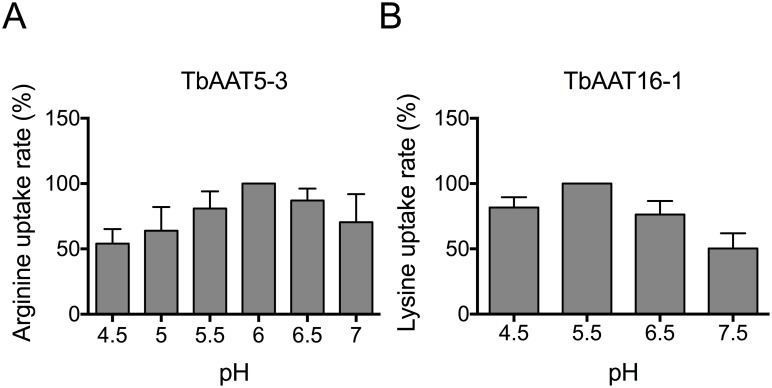
TbAAT5-3 mediated arginine and TbAAT16-1 mediated lysine transport are pH-dependent. (A) L-Arginine uptake rates in *S*. *cerevisiae* 21.983c expressing TbAAT5-3 were measured using 50 μM L-arginine and pH values between pH 4.5 and 7 (n = 3 ± SD). (B) TbAAT16-1-mediated 50 μM L-lysine transport at pH 4.5, 5.5, 6.5 and 7.5. Values correspond to the mean ± SD of at least seven independent experiments. Transport rates were determined as described in Materials and Methods. Relative transport rates were calculated by normalizing maximum values of each experiment to 100%.

Members of the AAAP family in plants are known to be driven by the proton motive force [50], while mammalian AAAP members may use either proton or sodium [51]. In general, uptake of different substrates in trypanosomes is proton-coupled, e.g. nucleosides [52], choline and *myo*-inositol [53,54], and amino acids [21]. Between pH 4.5 and 7, and using 50 μM L-arginine, the arginine uptake rates were only slightly pH-dependent, indicating that TbAAT5-3 may mediate arginine uptake in different pH environments (Fig 3A). TbAAT16-1 showed highest 50 μM L-lysine transport rates at pH 5.5. At pH 4.5 and 6.5 the relative transport rate was approximately 80% while at pH 7.5, lysine uptake was reduced to 50% (Fig 3B).

**Fig 4:**
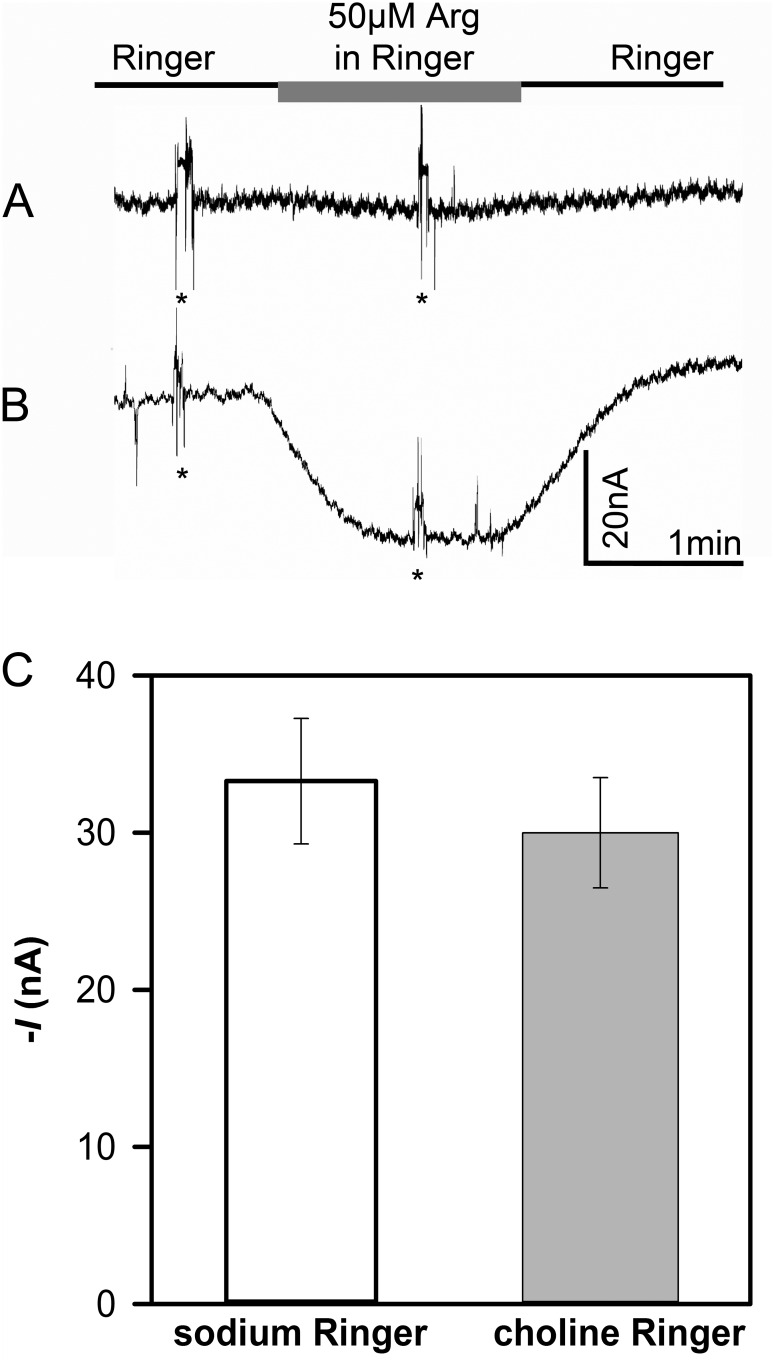
Arginine-induced currents in TbAAT5-3-expressing oocytes in the presence or absence of Na^+^ and K^+^. Oocytes were clamped at -80 mV. Inward currents were recorded upon superfusion of H_2_O-injected oocytes (A) and TbAAT5-1 injected oocytes (B) with Na^+^-Ringer containing 50 μM arginine at pH 7.4 (B). (C) Currents evoked by 20 μM arginine in sodium Ringer or choline Ringer (without sodium and potassium) at *V*_m_ of -80 mV. Mean values ± SE of 5 oocytes. Spikes in the current traces (marked by stars) are due to changes of solutions.

To determine the dependence on Na^+^ and K^+^, substrate-induced currents were analyzed in *Xenopus laevis* oocytes injected with TbAAT5-3 or TbAAT16-1 cRNA using two-electrode voltage clamping. While no currents could be recorded for *TbAAT16-1* when using up to 10 mM lysine, arginine or histidine as substrates (pH 7.4 and pH 5.5), in oocytes injected with *TbAAT5-3* cRNA, the addition of 50 μM arginine in Na^+^-Ringer induced small inward currents in the range of 15 to 90 nA (Fig 4B, *V*_m_ -80 mV, pH 7.4). Comparable currents were measured when Na^+^ and K^+^ were replaced by choline (choline Ringer, Fig 4C). These findings indicate that arginine transport via TbAAT5-3 does not depend on extracellular Na^+^ and K^+^.

### TbAAT5-3 and TbAAT16-1 mediate high affinity, selective transport of arginine and lysine, respectively

**Fig 5:**
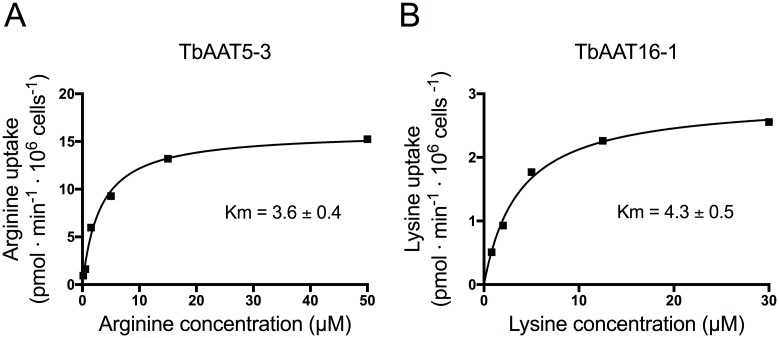
Transport kinetics of TbAAT5-1 and TbAAT16-1. Kinetics of arginine (A) or lysine (B) uptake by TbAAT5-1 or TbAAT16-1 expressing *S*. *cerevisiae* strain 21.983c or 22Δ7AA, respectively. Representative graphs are shown. Affinities (*K*_m_) are mean values ± SD of three independent experiments.

Transport assays performed using *S*. *cerevisiae* expressing TbAAT5-3 revealed high affinity transport of L-[^3^H]-arginine, with a *K*_m_ of 3.6 ± 0.4 μM (strain 21.983c, Fig 5A), while expression of TbAAT16-1 showed high affinity transport of L-[^3^H]-lysine, with a *K*_m_ of 4.3 ± 0.5 μM (strain 22Δ7AA, Fig 5B).

Competition of TbAAT5-3 mediated (50 μM) L-arginine uptake showed that transport was only reduced by a 10 times excess of L-arginine, canavanine, homo-arginine and arginine-β-naphthylamide, but not by 10 or 100 times excess of citrulline, agmatine, cadaverine, histidine or ornithine, or other arginine analogs tested (Table 1). D-arginine did not inhibit L-arginine uptake, indicating that transport of L-arginine by TbAAT5-3 is stereospecific. Although TbAAT5-3 mediated growth on histidine and lysine, competition for L-arginine transport was only observed at 100 times excess of lysine (Table 1). Similarly, 50 μM L-lysine uptake mediated by TbAAT16-1 was only inhibited by a 10-times excess of L-lysine, Fmoc-lysine and thialysine (Table 2). Alanine, acetyl-L-lysine, arginine, aspartate, eflornithine, histidine, D-lysine, hydroxy-L-lysine, methyl-L-lysine, the dipeptide lysine-lysine, ornithine or proline did not compete for L-lysine transport (Table 2).

**Table 1:** Substrate selectivity of TbAAT5-3.

TbAAT5-3	L-[^3^H]-arginine uptake rate (%)	
Competitor added	10x	100x
None	100	100
L-Arginine	9.9 ± 2.8	n.d.
D-Arginine	124.2 ± 6	n.d.
Histidine	107.5 ± 7.3	93.5 ± 7.8
Lysine	93.6 ± 6.2	56.5 ± 8.2
Citrulline	112.4 ± 10.9	114.2 ± 6.3
Canavanine	38.5 ± 4.5	7.6 ± 5.9
Homoarginine	25.6 ± 4.2	8 ± 6.2
Ornithine	96.6 ± 12.7	91.9 ± 24.5
Arginine methyl ester	116.1 ± 15.6	67.6 ± 10.3
Arginine ethyl ester	129.4 ± 5.8	72.5 ± 8.7
Arginine-β-naphthylamide	69 ± 6.6	16.6 ± 10
Tosyl-arginine	94 ± 3.7	n.d.
Arginine-tosylate	86.7 ± 17.4	n.d.
ADMA	104.4 ± 14.2	n.d.
L-NMMA	77.8 ± 11.5	n.d.
Agmatine	n.d.	105.4 ± 20.6
Cadaverine	n.d.	115 ± 5.8

50 μM L-[^3^H]-arginine uptake rates were measured in *S*. *cerevisiae* strain 21.983c expressing TbAAT5-3, in the presence of 500 μM or 5 mM of various compounds (pH 6.0). Data was normalized on arginine transport rates in the absence of competitors (100%). Transport rates varied between 0.3–40 pmol min^-1^ 10^6^ cells^-1^. Mean of at least three independent experiments ± SD are shown. L-NMMA, *N*^*G*^-monomethylarginine; ADMA, *N*^G^,*N*^G^-dimethylarginine. If not indicated otherwise, the L-form of the amino acid was used.

**Table 2:** Substrate selectivity TbAAT16-1.

TbAAT16-1	L-[^3^H]-lysine uptake rate (%)
Competitor added	10x
None	100
L-Lysine	4.6 ± 1.7
D-Lysine	104.6 ± 11.5
Histidine	94.6 ± 15.4
Arginine	107.9 ± 9.6
Proline	101.5 ± 2.6
Aspartate	106.2 ± 8.3
Alanine	104.4 ± 6.7
Ornithine	95.2 ± 9.9
Eflornithine	96.1 ± 6.0
Fmoc-Lysine	57.6 ± 6.9
Methyl-L-lysine	82.9 ± 9.2
Acetyl-L-lysine	95.7 ± 9.4
Hydroxy-L-lysine	88.0 ± 8.1
Thialysine	35.3 ± 1.8
Lysine-lysine	98.7 ± 11.2

50 μM L-[^3^H]-lysine uptake rates were measured in *S*. *cerevisiae* expressing TbAAT16-1 (22Δ7AA strain), in the presence of 500 μM of various compounds at pH 4.5. Data were normalized relative to lysine uptake rates in the absence of competitors (100%). Transport rates varied between 0.5–7.8 pmol min^-1^ 10^6^ cells^-1^. Means of at least three independent experiments ± SD are shown. Fmoc-lysine, N-α-(9-fluorenylmethyloxycarbonyl)-L-lysine. If not indicated otherwise, the L-form of the amino acid was used.

### TbAAT5 and TbAAT16 are highly expressed and localized at the plasma membrane

**Fig 6:**
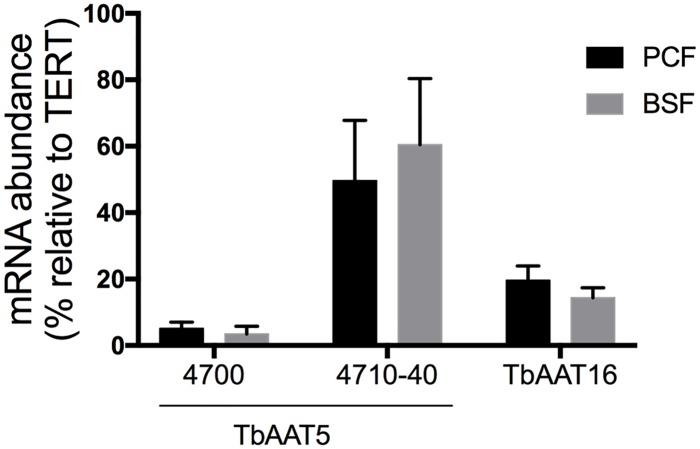
Expression of *TbAAT5* and *TbAAT16* members in PCF and BSF *T*. *brucei*. *TbAAT5* and *TbAAT16* mRNA abundance in PCF and BSF *T*. *brucei* was quantified by qRT-PCR using telomerase reverse transcriptase (TERT, Tb927.11.10190) as reference gene. Due to high homology of ORFs and UTRs (S1 Fig) only Tb427.08.4700 could be discriminated from the other *TbAAT5* members (i.e. Tb427.08.4710, Tb427.08.4720, Tb427.08.4730, Tb427.08.4740). The two *TbAAT16* genes (Tb427tmp.01.7500 and Tb427tmp.01.7520) were not differentiated.

**Fig 7:**
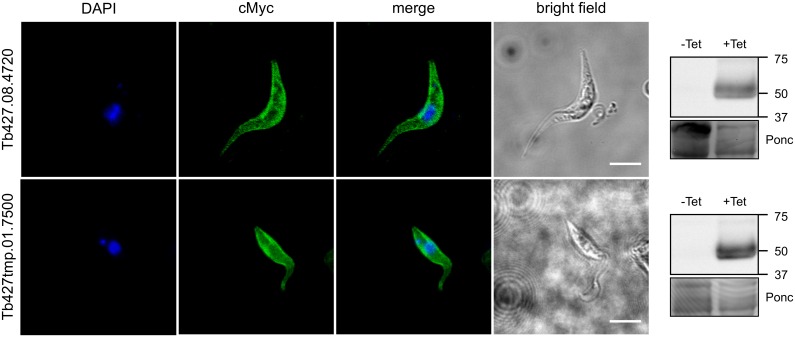
Localization of cMyc-TbAAT5-1 and cMyc-TbAAT16-1 in PCF *T*. *brucei*. Confocal microscopy of procyclic *T*. *brucei* cells over-expressing N-terminally cMyc-tagged versions of TbAAT5-1 and TbAAT16-1. The proteins localized in a bright ring along the periphery of the cell body and in internal membranes, showing partial localization at the plasma membrane as well as in internal membranes of unknown identity. Anti-cMyc (green); DAPI (blue); bright field image (grey); scale bar: 5 μM. The insets show Western blot analyses of extracts from uninduced (-tet) or induced (+tet) cells probed with anti-cMyc antibody. Ponceau (Ponc) staining is shown as loading control.

*TbAAT5* and *TbAAT16* transcripts were abundant in both PCF and BSF *T*. *brucei* (Fig 6). Due to high sequence homology only Tb427.08.4700, which has a distinct 3’UTR, could be distinguished from the other *TbAAT5* members (see S1 Fig). Transcriptome analyses support high transcript levels of *TbAAT5* and *TbAAT16* in BSF and PCF trypanosomes [45], in line with *T*. *brucei* being auxotroph for cationic amino acids [5], and thus, depending on their import in insect and mammalian hosts. While protein synthesis necessitates lysine and arginine import into the cytosol and the mitochondrion, the presence of arginine kinase in the cytosol, glycosome and flagellum [14] indicates the presence of arginine in additional subcellular compartments. Protein sorting motifs are generally better defined for soluble proteins than for membrane proteins and the fact that none of the sorting motif for mitochondria (using MitoProt [55] or TargetP [56]), peroxisomes/glycosomes [57] or flagellum [58] could be identified, does not exclude localization in the membrane of these subcellular compartments. Proteome analyses did not identify these transporters in the flagellum [59,60] or glycosome proteomes [61–63]. PCF *T*. *brucei* over-expressing cMyc-tagged versions of either TbAAT5-3 or TbAAT16-1 indicated plasma membrane localization of the tagged proteins (Fig 7), which is consistent with TbAAT5 or TbAAT16 mediating import of extracellular arginine or lysine, respectively, in *S*. *cerevisiae*. Fluorescence was also detected in unknown intracellular structures, possibly as a result of overexpression of the tagged protein.

### TbAAT5 and TbAAT16 are essential for arginine and lysine uptake and growth of bloodstream form trypanosomes

**Fig 8:**
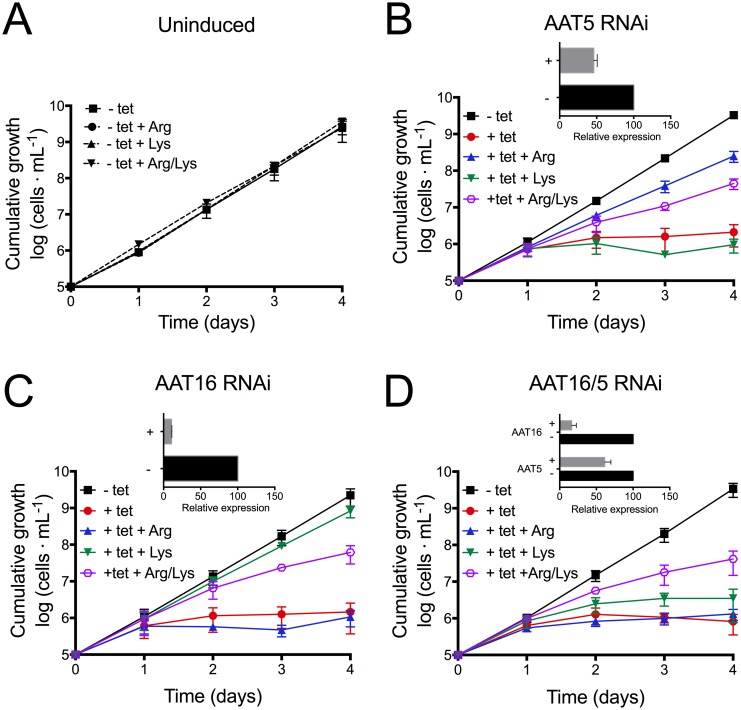
Growth curves of TbAAT5-RNAi and TbAAT16-RNAi *T*. *brucei* BSF. Cumulative growth of *T*. *brucei* BSF control (A) or after down-regulation of *TbAAT5* (B), *TbAAT16* (C) or both (D) for 5 days in HMI-9. 5 mM arginine and/or 10 mM lysine was added as indicated above the growth curves. Data points of growth curves correspond to mean values ± SD from three independent experiments. Insets show mRNA levels relative to telomerase reverse transcriptase (TERT, Tb927.11.10190) transcripts after one day of induction as determined by qRT-PCR. Expression in the absence of tetracycline was set as 100%. Data points correspond to mean values ± SD from three independent experiments.

To investigate the role of TbAAT5 and TbAAT16 in trypanosomes, expression was down-regulated by RNAi using a tetracycline-inducible system [36]. After one day of induction, mRNA of TbAAT5 and TbAAT16 were significantly reduced. RNAi against *TbAAT5* or *TbAAT16* substantially reduced growth of BSF parasites after one day of induction (Fig 8B and 8C), thus hampering mRNA isolation at later time points.

Although down-regulation of individual genes was not analyzed, the high homology among the 5 genes of the *AAT5* locus (> 98% overall identity on nucleotide level) and between the 2 genes of the *AAT16* locus (99% overall identity on nucleotide level) suggests that the RNAi constructs targeted all genes of the *TbAAT5* or *TbAAT16* locus, respectively.

To test the hypothesis that the growth defect of the induced RNAi lines is due to the inability to import sufficient amounts of arginine or lysine, the medium was supplemented with 5 mM arginine or 10 mM lysine, i.e. approximately 10 times the concentration of arginine or lysine in HMI-9 (482 μM Arg and 1 mM Lys, respectively [64]). Indeed, the addition of arginine or lysine to the medium significantly improved growth of the tetracycline-induced *TbAAT5*-RNAi (Fig 8B) or *TbAAT16*-RNAi (Fig 8C) BSF *T*. *brucei*, respectively, while growth of non-induced cells remained unchanged (Fig 8A). In contrast, addition of both amino acids improved growth of induced RNAi lines less efficiently, indicating that at least part of the import at high substrate concentrations is mediated by a system that transports both arginine and lysine. Down-regulation of *TbAAT5* in PCF parasites also impacts growth indicating that arginine uptake is essential in this life-cycle stage (S2 Fig). In a double-RNAi BSF line (TbAAT5/16-RNAi) with simultaneous down-regulation of *TbAAT5* and *TbAAT16*, reduction of transcript levels was comparable to the single RNAi lines (Fig 8D). Induced TbAAT5/16-RNAi parasites stopped growth after one day of tetracycline addition. As expected, supplementing the medium with 5 mM arginine or 10 mM lysine did not prevent the growth defect. Addition of both arginine and lysine resulted in partial rescue, comparable to the addition of both amino acids to single RNAi knockdown lines.

## Discussion

In mammalian cells the cationic amino acids arginine and lysine are transported by the same transport systems that mediate exchange of cationic amino acids and cysteine against intracellular neutral amino acids (rBAT), or facilitated uptake of cationic amino acids (CAT) [26,65]. CAT members have similar affinities for arginine, lysine and ornithine (*K*_m_ 40–450 μM for CAT1, CAT2B and CAT3; 2–5 mM for CAT2A) [27] while histidine recognition varies. In contrast, the cationic amino acid transporters characterized from the parasitic protozoa *L*. *donovani* and *T*. *cruzi* are selective for either arginine (LdAAP3, TcAAAP411) or lysine (LdAAP7, TcAAP7) and belong to a different gene family [22–24]. Only TcCAT1.1 has recently been described as arginine/ornithine transporter [25], but the huge difference in affinity for the two substrates indicates that the latter may not be the preferred substrate *in vivo*. Our results show that similar to the trypanosomatid transporters mentioned above, uptake of cationic amino acids in *T*. *brucei* is mediated by selective and high affinity transporters.

Despite the similarities among trypanosomatid arginine and lysine transporters, subtle differences were detected. At saturating arginine concentrations, L-arginine transport mediated by TbAAT5-3 was not affected by the presence of a 10 times excess of the cationic amino acids histidine, ornithine, and lysine, with the latter partially reducing arginine transport only at 100 times excess, indicating high selectivity, comparable to LdAAP3 and TcCAT1.1 [22,25]. Furthermore, like the arginine transporter TcAAAP411 [23], TbAAT5-3 is stereoselective since D-arginine has no effect on arginine transport. The arginine analogs canavanine and homoarginine were inhibitors of TbAAT5-mediated arginine transport, suggesting that the aliphatic chain between the guanidino group and the amino- and the carboxylic acid-group is less important for substrate recognition. Canavanine was also found to be a good competitor for TcCAT1.1-mediated arginine uptake, while homoarginine was less efficient [25]. In contrast, in TcAAAP411-expressing *S*. *cerevisiae* cells, canavanine was competing only at 50-fold excess, and for LdAAP3 homoarginine at 5-fold excess did not inhibit arginine uptake [22,23], revealing slight preferences in substrate recognition. The arginine analogs homoarginine and canavanine were not only competitors in heterologous expression systems, but were also the best competitors for arginine uptake in *T*. *cruzi* epimastigotes [66]. Interestingly in *T*. *cruzi* the arginine analogs agmatine, homoarginine and canavanine were shown to inhibit arginine kinase and the latter two compounds were also able to reduce *T*. *cruzi* epimastigote growth in culture [67].

Agmatine neither interfered with TbAAT5-3 nor with LdAAP3- or TcCAT1.1-mediated arginine transport indicating that the negatively charged carboxylic acid is essential for substrate recognition [22,23]. Furthermore, alkylation of the carboxylic acid moiety of arginine also decreased competition for arginine uptake by TbAAT5-3 as indicated by the much smaller reduction of arginine transport rates by arginine ethyl ester and arginine methyl ester that diminished arginine transport rates to about 70% (at 100 times excess). In addition to the lack of a negative charge in these compounds, steric constraints might explain reduced interaction of arginine derivatives esterified at the carboxylic acid group. In contrast, arginine β-naphthylamide reduced arginine transport rates, though it was not tested whether the amphipathic compound may reduce transport through an unspecific effect.

Methylation at the guanidine group of arginine lowered recognition by TbAAT5-3 substantially as seen for *N*^*G*^-monomethylarginine (L-NMMA). A second methylation of the same amino group in *N*^G^,*N*^G^-dimethylarginine (ADMA) completely impaired recognition by TbAAT5-3. And finally, a guanidine group is preferred to an amine group as lysine only decreases arginine transport if present at 100 times excess. Comparably, the affinity of *Leishmania* LdAAP3 for lysine was about 500 times lower than for arginine [22].

The lysine transporter, Tb427tmp.01.7500, is also highly selective. Similar to LdAAP7-mediated lysine transport and lysine uptake into *T*. *cru*zi epimastigotes and *L*. *donovani* promastigotes, none of the proteinogenic amino acids tested competed for L-lysine uptake [24]. Transport was stereospecific and modifications of the amino group (N-α-acetyl-L-lysine methyl ester), shortening the length of the carbon scaffold as in ornithine and eflornithine (α-difluoromethylornithin) or extending it at the ε-amino residue (N-ε-methyl-L-lysine) impaired recognition as substrates. Also the dipeptide lysyllysine was not recognized. While hydroxylation of the carbon backbone (5-hydroxy-L-lysine) was not tolerated, thialysine (S-(2-aminoethyl)-L-cysteine) in which C2 is replaced by a sulphur was the only compound competing for lysine uptake and, to a lesser extend, Fmoc-lysine (N-α-(9-fluorenylmethyloxycarbonyl)-L-lysine) also reduced L-lysine uptake rates. The various compounds were tested to evaluate the requirements for a substrate to be recognized by TbAAT5 and TbAAT16. In the future, structural models of the transporters may allow identification of the residues involved in substrate binding and selectivity.

The apparent affinities of TbAAT5-3 (*K*_m_ 3.6 μM) and TbAAT16-1 (*K*_m_ 4.3 μM) for arginine and lysine, respectively, indicate that the transporters are likely to be saturated and working at maximal rates in mammalian blood where concentrations of 20–218 μM arginine and 164–434 μM lysine have been reported (Human metabolome database, www.hmdb.ca [68]). In human cerebrospinal fluid, 9–25 μM arginine and 22–32 μM lysine were found [68], suggesting that the transporters also mediate uptake of these amino acids in *T*. *brucei* parasites in the central nervous system. Although TbAAT5 is more closely related to TcAAAP411 and TcCAT1.1 (Fig 1), the apparent affinity of TbAAT5 for arginine determined in *S*. *cerevisiae* is close to 10 times (TcAAAP4111) and more than 20 times (TcCAT1.1) higher and comparable to the *K*_m_ determined for the arginine-selective transport system AAP3 from *L*. *donovani* [22,23,25]. All trypanosomatid lysine transporters characterized so far are highly homologous, and also the *K*_m_ values were in a comparable range [24]. While transport of arginine and lysine into *T*. *brucei* parasites has not been determined, the affinity of lysine and arginine uptake *in L*. *donovani* promastigotes (*K*_m_ values of 3 μM and 14 μM, respectively) are in a range comparable to the *K*_m_ value determined in *S*. *cerevisiae* expressing LdAAP7 (7.4 ± 3.6 μM) or LdAAP3 (1.9 ± 0.1 μM) [22,24,69] and similar to the affinity of the *T*. *brucei* lysine and arginine transporters characterized in our study. Lysine transport assessed in *T*. *cruzi* epimastigotes revealed a slightly lower affinity of 23.4 ± 2.3 μM, while kinetic data of TcAAP7 expressed in *S*. *cerevisiae* were not determined [24].

Down-regulation of *TbAAT5* and *TbAAT16* resulted in impaired growth of *T*. *brucei* parasites, confirming their importance for arginine and lysine uptake. The growth defect could only be rescued at very high, i.e. non-physiological concentrations of arginine or lysine, respectively. Initially, growth phenotypes of the *S*. *cerevisiae* mutants expressing TbAAT5 or TbAAT16 indicated that the transporters recognize both arginine and lysine. However, the competition experiments showed that with a 10-fold excess there is no reduction of transport rates, and thus *in vivo*, with concentrations of arginine and lysine differing ~ 2-fold, transport of both amino acids by the same transporters is very unlikely. Furthermore, the reduction of growth of *TbAAT5* RNAi lines in the presence of high arginine and high lysine compared to high arginine indicated that uptake is mediated partially by a system that also recognizes lysine. This transport system is, however, unlikely to be TbAAT16, because lysine concentrations in HMI9 medium are at saturation for TbAAT16 and a 10-fold excess of arginine is not expected to be transported at high rates. The same line of arguments can be used for the *TbAAT16* RNAi lines grown in the presence of lysine or lysine plus arginine. Single and double RNAi lines therefore indicate that additionally one or several low affinity transport systems are present and that at least one of them recognizes both arginine and lysine. Low affinity arginine transport has so far only been described in *T*. *cruzi* [70], but the corresponding gene has not been identified and low affinity lysine or arginine transport has not been determined in *T*. *brucei*.

Dependence of parasites on these high affinity lysine and arginine transport systems is likely to be more important *in vivo* since—as indicated above—the concentrations of cationic amino acids in the blood are 5–6 times lower and in the cerebrospinal fluid 30-70-times lower than in standard HMI-9 medium [64,68]. Understanding the multiple mechanisms by which the parasites acquire nutrients remains of major importance to comprehend parasite physiology and may help developing new trypanocidal compounds. TbAAT5 and TbAAT16 may be promising candidates as drug delivery systems or targets, because they are essential and not related to human cationic amino acid uptake systems.

## Supporting Information

S1 Table Comparison of *T*. *brucei AAT5*-genes from strains TREU927 and 427 with ORFs amplified by PCR. The nucleotide positions are according to the annotated sequences of *AAT5* genes in strain TREU927 in the TriTrypDB, containing 1377 nucleotides. Changes in amino acids are indicated when different from the reference gene Tb427.08.4720 (boxed in red). Conserved nucleotides in strain 427 and TREU927 are highlighted in blue or orange. N, unresolved nucleotides in strain 427; del, predicted deletion in Tb427.8.4700. G/C* nucleotide (G) present in the primer and not verified by PCR.
(PDF)

S2 Table Percentage of identity and similarity of predicted amino acid sequences of *T*. *brucei* AAT5 and AAT16 members and characterized arginine and lysine transporters from *T*. *cruzi* and *Leishmania*. Percentage of identity and similarity of members of the AAT5 and AAT16 family of *T*. *brucei brucei* strain TREU927, characterized arginine and lysine transporters from *T*. *cruzi* and *L*. *donovani*: TcAAAP411, (TcCLB.511411.30 [23]), TcAAP7 (TcCLB.511545.80 [24]), TcCAT1.1, TcCAT1.2, TcCAT1.3 (TcCLB.506153.10, TcCLB.506153.20, TcCLB.506053.10 [25]); LdAAP7 (LdBPK_322800.1 [24]) and LdAAP3 (LdBPK_310910.1 [22]). Included are also *L*. *major* AAP7 (LmjF.32.2660); and the characterized, but more distantly related transporter of eflornithine and neutral amino acids, TbAAT6 (Tb927.8.5450, [21]). Because the ORF of TcCLB.506053.5 is much shorter, it was not included.
(TIF)

S3 Table Comparison of *T*. *brucei AAT16*-genes from strains 427 and TREU927 with ORFs amplified by PCR. The predicted ORFs have the same length in strain TREU927 and 427 (ORF 1389 nt, TriTrypDB). Changes in amino acids are indicated when different from Tb427tmp.01.7500 (boxed in red). *The amino acid sequence of Tb427tmp.01.7520 was generated by mutagenesis of T^1028^ in Tb427tmp.01.7520_G343V to G, thereby changing valine to glycine. Conserved nucleotides found in strain 427 and TREU927 are highlighted in blue or orange. N, unresolved nucleotides in strain 427.
(PDF)

S1 Fig Organization of *AAT5* and *AAT16* loci in the *T*. *brucei* genome. (A) The 6 copies of the *TbAAT5* locus of *T*. *brucei* are located on chromosome 8, i.e. Tb427.08.4700, Tb427.08.4710, Tb427.08.4720, Tb427.08.4730, Tb427.08.4740, and the 120 nt long pseudogene Tb427.08.4750. (B) The two copies of the *TbAAT16* locus are found on chromosome 11, i.e. Tb427tmp.01.7500 and Tb427tmp.01.7520. Open reading frames (ORFs) and untranslated regions (UTRs) are based on the annotation in the reference strain TREU927. Regions used for qRT-PCR on RNA isolated from RNAi clones (to assess *TbAAT5* or *TbAAT16* down-regulation) or from parental cell lines (to compare transcript levels in BSF vs PCF) are indicated. UTR sequences are color-coded with similar colors representing high identity. Schemes are based on the TriTrypDB annotation (see http://tritrypdb.org).
(PDF)

S2 Fig Growth curve of *TbAAT5*-RNAi PCF *T*. *brucei*. Growth of *T*. *brucei* procyclic forms after down-regulation of *TbAAT5* by RNAi. Data points are mean values of three independent experiments ± SD. The inset shows Northern blot analysis of total RNA extracted from trypanosomes after 2 days of incubation in the absence (-) or presence (+) of tetracycline (Tet) and probed with ^32^P-labeled oligonucleotide fragments used as inserts for the respective stem-loop vectors. Ethidium bromide (EtBr) staining is shown as loading control. Northern blot was performed as described previously [53].
(TIFF)
