# Supplementary material for: Arginine and Lysine Transporters Are Essential for Trypanosoma brucei
Source: PLoS One. 2017 Jan 3;12(1):e0168775. doi: 10.1371/journal.pone.0168775 (PMC5207785; doi:10.1371/journal.pone.0168775)
Supplement: S1 Table — The nucleotide positions are according to the annotated sequences of AAT5 genes in strain TREU927 in the TriTrypDB, containing 1377 nucleotides. Changes in amino acids are indicated when different from the reference gene Tb427.08.4720 (boxed in red). Conserved nucleotides in strain 427 and TREU927 are highlighted in blue or orange. N, unresolved nucleotides in strain 427; del, predicted deletion in Tb427.8.4700. G/C* nucleotide (G) present in the primer and not verified by PCR. (PDF) [file pone.0168775.s001.pdf]

	ORFs in TriTrypDB																			ORFs independently amplified by PCR					
	strain 927										strain 427									strain 427					
**Nucleotide position in 927**	**Tb927.8.4710**	**aa change**	**Tb927.8.4720**	**aa change**	**Tb927.8.4730**	**aa change**	**Tb927.8.4700**	**aa change**	**Tb927.8.4740**	**aa change**	**Tb427.08.4710**	**aa change**	**Tb427.08.4720**	**Tb427.08.4730**	**aa change**	**Tb427.08.4700**	**aa change**	**Tb427.08.4740**	**aa change**	**Tb427.08.4720 (AAT5-3)**	**aa change**	**Tb427.08.4720_D20N**	**aa change**	**Tb427.08.4740**	**aa change**
6	G		G		G		G		G		C		C	C		C		C		*G/C**		*G/C**		*G/C**	
19	G	**P to A**	C		G	**P to A**	C		C		**Ns (19-24)**		C	**Ns (19-25)**		C		C		C		C		C	
24	G		A		G		A		A				A			A		A		A		A		A	
58	G		G		G		G		G		G		G	G		G		G		G		A	**D to N**	G	
60-63	TGAG		TGAG		TGAG		TGAG		TGAG		TGAG		TGAG	TGAG		**del 60-63**		TGAG		TGAG		TGAG		TGAG	
96	C	D	C	D	C	D	C	D	C	D	C	D	**N**	**N**		**N**		**N**		C	D	C	D	G	**D to E**
121	A		G		A		G		G		G		G	G		G		G		G		G		G	
128	T	**P to L**	C		T	**P to L**	C		C		C		C	T	**P to L**	C		C		C		C		C	
183	T		T		T		T		T		C		C	C		C		C		C		C		C	
273	T		C		T		C		C		T		C	C		C		C		C		C		C	
369	T		T		T		C		C		T		T	T		C		C		T		T		C	
370	C		C		C		A	**A to T**	A	**A to T**	G		G	G		A	**A to T**	A	**A to T**	G		G		A	**A to T**
486	G		G		G		C		C		G		G	G		C		C		G		G		C	
488	A		A		A		G	**D to G**	G	**D to G**	A		A	A		G	**D to G**	G	**D to G**	A		A		G	**D to G**
495	G		G		G		T		T		G		G	G		T		T		G		G		T	
496	C		C		C		G	**L to A**	G	**L to A**	C		C	C		G	**L to A**	G	**L to A**	C		C		G	**L to A**
497	T		T		T		C		C		T		T	T		C		C		T		T		C	
498	A		A		A		T		T		A		A	A		T		T		A		A		T	
506	C		C		C		G	**S to C**	G	**S to C**	C		C	C		G	**S to C**	G	**S to C**	C		C		G	**S to C**
508	T		T		T		A	**S to T**	A	**S to T**	T		T	T		A	**S to T**	A	**S to T**	T		T		A	**S to T**
511	G		G		G		A	**A to T**	A	**A to T**	G		G	G		A	**A to T**	A	**A to T**	G		G		A	**A to T**
618	T		T		T		T		T		C		C	C		C		C		C		C		C	
936	C		T		C		T		T		T		T	C		T		T		T		T		T	
1126	T	**S to C**	A	**S to C**	T	**S to C**	T	**S to C**	T	**S to C**	T		T	T		T		T		T		T		T	
