# Supplementary figures and images for: Arginine and Lysine Transporters Are Essential for Trypanosoma brucei
Source: PLoS One. 2017 Jan 3;12(1):e0168775. doi: 10.1371/journal.pone.0168775 (PMC5207785; doi:10.1371/journal.pone.0168775)

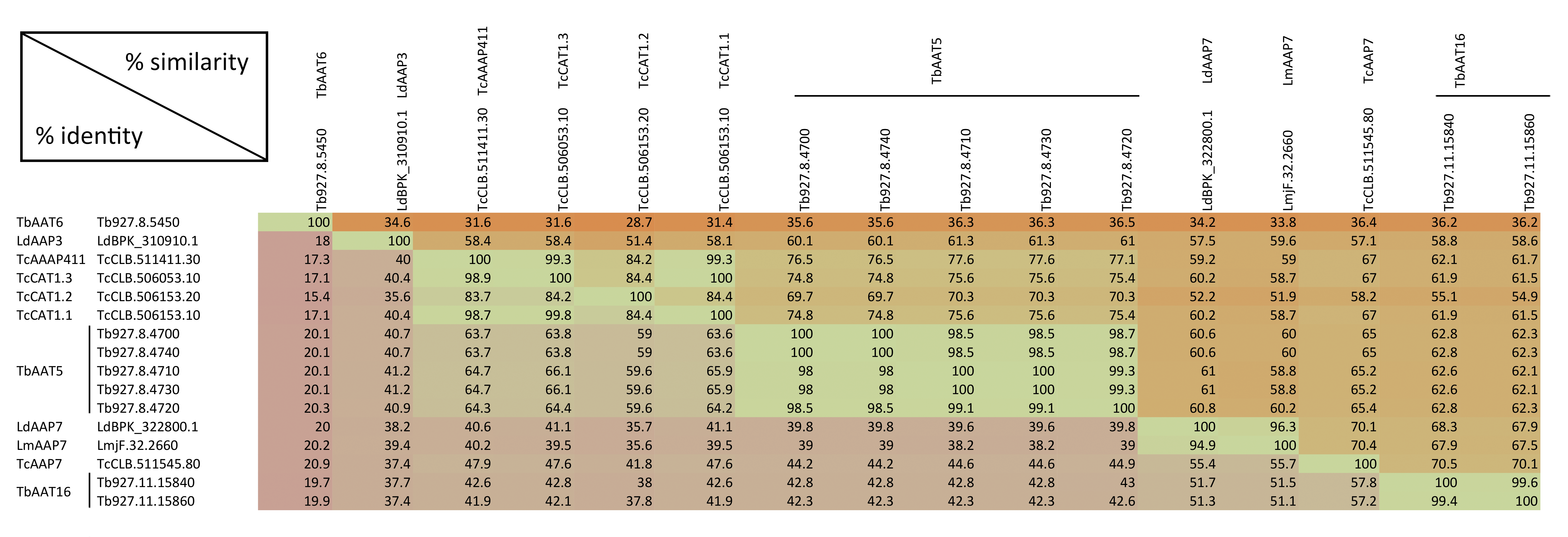

Supplement: S2 Table — Percentage of identity and similarity of members of the AAT5 and AAT16 family of T. brucei brucei strain TREU927, characterized arginine and lysine transporters from T. cruzi and L. donovani: TcAAAP411, (TcCLB.511411.30 [23]), TcAAP7 (TcCLB.511545.80 [24]), TcCAT1.1, TcCAT1.2, TcCAT1.3 (TcCLB.506153.10, TcCLB.506153.20, TcCLB.506053.10 [25]); LdAAP7 (LdBPK_322800.1 [24]) and LdAAP3 (LdBPK_310910.1 [22]). Included are also L. major AAP7 (LmjF.32.2660); and the characterized, but more distantly related transporter of eflornithine and neutral amino acids, TbAAT6 (Tb927.8.5450, [21]). Because the ORF of TcCLB.506053.5 is much shorter, it was not included. (TIF) [file pone.0168775.s002.tif]

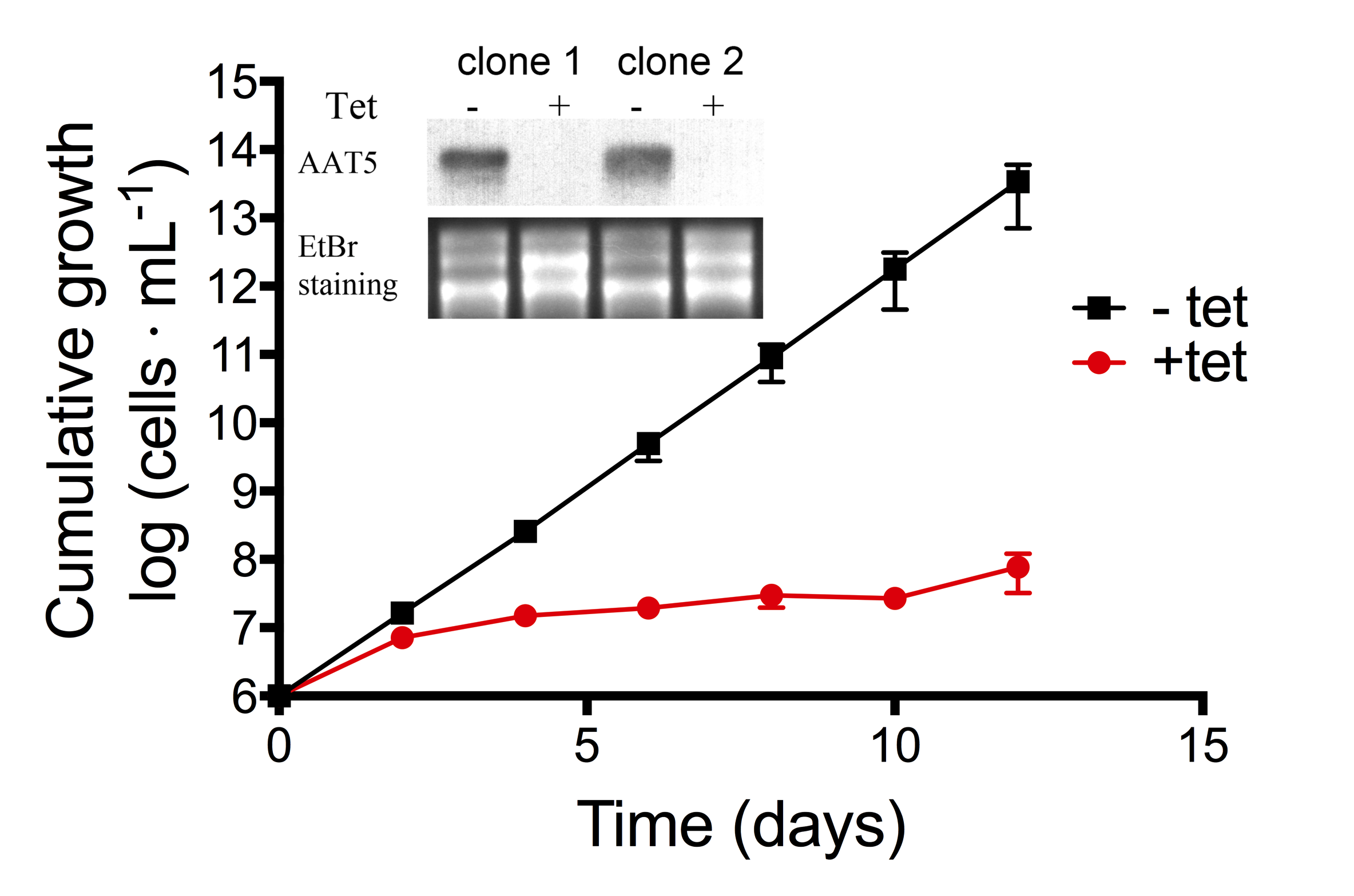

Supplement: S2 Fig — Growth of T. brucei procyclic forms after down-regulation of TbAAT5 by RNAi. Data points are mean values of three independent experiments ± SD. The inset shows Northern blot analysis of total RNA extracted from trypanosomes after 2 days of incubation in the absence (-) or presence (+) of tetracycline (Tet) and probed with 32P-labeled oligonucleotide fragments used as inserts for the respective stem-loop vectors. Ethidium bromide (EtBr) staining is shown as loading control. Northern blot was performed as described previously [53]. (TIFF) [file pone.0168775.s005.tiff]
