# Supplementary material for: Arginine and Lysine Transporters Are Essential for Trypanosoma brucei
Source: PLoS One. 2017 Jan 3;12(1):e0168775. doi: 10.1371/journal.pone.0168775 (PMC5207785; doi:10.1371/journal.pone.0168775)
Supplement: S3 Table — The predicted ORFs have the same length in strain TREU927 and 427 (ORF 1389 nt, TriTrypDB). Changes in amino acids are indicated when different from Tb427tmp.01.7500 (boxed in red). *The amino acid sequence of Tb427tmp.01.7520 was generated by mutagenesis of T1028 in Tb427tmp.01.7520_G343V to G, thereby changing valine to glycine. Conserved nucleotides found in strain 427 and TREU927 are highlighted in blue or orange. N, unresolved nucleotides in strain 427. (PDF) [file pone.0168775.s003.pdf]

	ORFs in TriTrypDB							ORFs amplified by PCR						Generated by mutagenesis	
	strain 927				strain 427			strain 427							
Nucleotide position in 927	Tb927.11.15840	aa change	Tb927.11.15860	aa change	Tb427tmp.01.7500	Tb427tmp.01.7520	aa change	Tb427tmp.01.7500 (AAT16-1)	aa change	Tb427tmp.01.7500_V343G	aa change	Tb427tmp.01.7520_G343V	aa change	Tb427tmp.01.7520	aa change
60	A		A		C	C		C		C		C		C	
105	T		T		T	T		T		T		C		C	
111	T		T		T	T		T		T		C		C	
153	G		G		G	N		G		G		T		T	
175	A		G	I to V	A	G	I to V	A		A		G	I to V	G	I to V
183	A		G		A	G		A		A		G		G	
185	A		C	Y to S	A	C	Y to S	A		A		C	Y to S	C	Y to S
411	T		T		C	C		C		C		C		C	
531	C		C		T	T		T		T		T		T	
747	A		A		G	G		G		G		G		G	
1023	A		T		A	T		A		T		A		A	
1026	C		T		C	T		C		T		C		C	
1028	T		G	V to G	T	G	V to G	T		G	V to G	T		G	V to G
1029	A		G		A	G		A		G		A		A	
1032	A		G		A	G		A		G		A		A	
1230	T		T		N	N		G		T		G		G	
1335	A		A		G	G		G		A		A		A	
1389	G		G		A	A		G		G		G		G	
