# Supplementary material for: Arginine and Lysine Transporters Are Essential for Trypanosoma brucei
Source: PLoS One. 2017 Jan 3;12(1):e0168775. doi: 10.1371/journal.pone.0168775 (PMC5207785; doi:10.1371/journal.pone.0168775)

AAT5 locus region (Chr. 8)

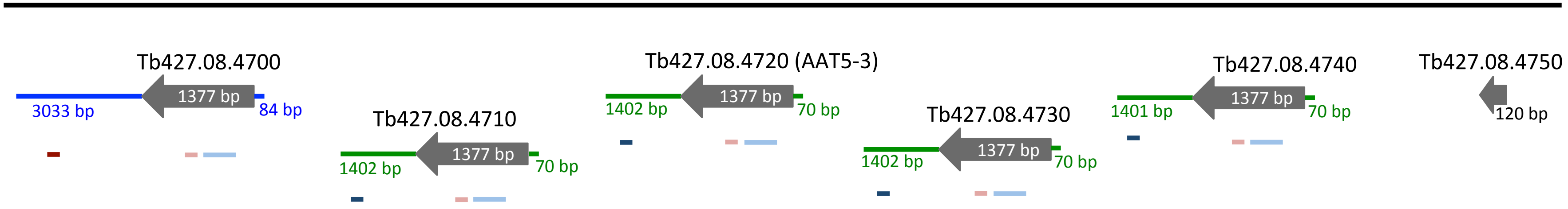

AAT16 locus region (Chr. 11)

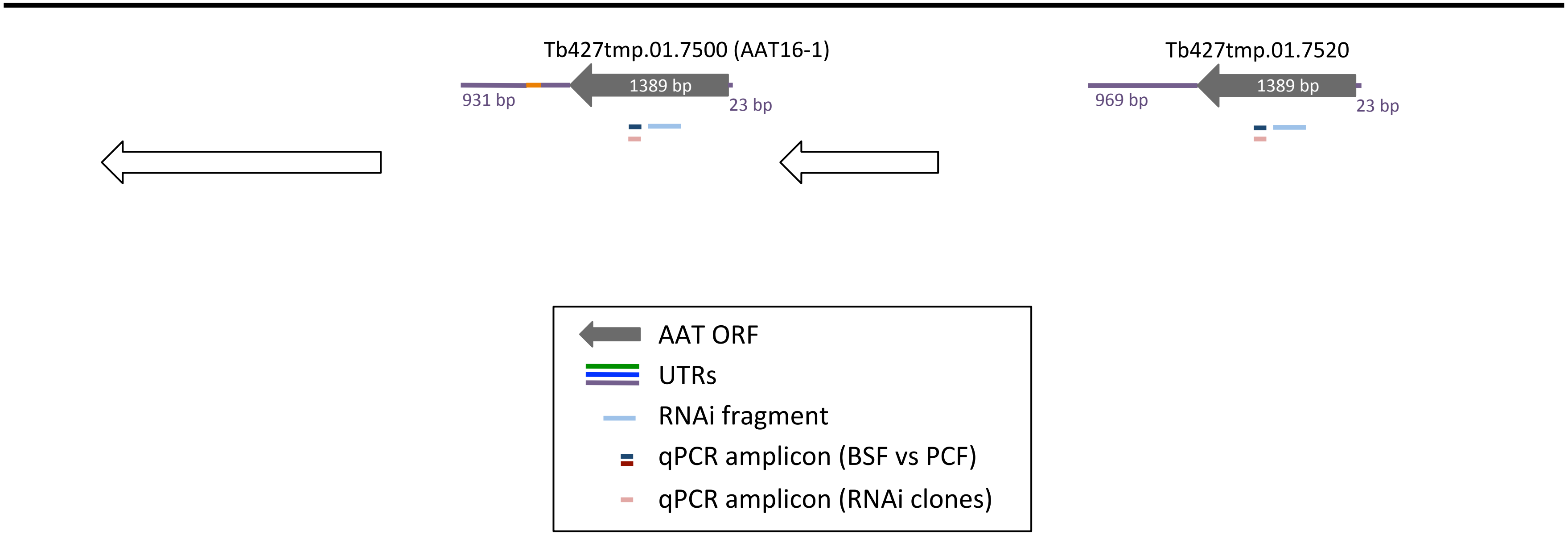

Supplement: S1 Fig — (A) The 6 copies of the TbAAT5 locus of T. brucei are located on chromosome 8, i.e. Tb427.08.4700, Tb427.08.4710, Tb427.08.4720, Tb427.08.4730, Tb427.08.4740, and the 120 nt long pseudogene Tb427.08.4750. (B) The two copies of the TbAAT16 locus are found on chromosome 11, i.e. Tb427tmp.01.7500 and Tb427tmp.01.7520. Open reading frames (ORFs) and untranslated regions (UTRs) are based on the annotation in the reference strain TREU927. Regions used for qRT-PCR on RNA isolated from RNAi clones (to assess TbAAT5 or TbAAT16 down-regulation) or from parental cell lines (to compare transcript levels in BSF vs PCF) are indicated. UTR sequences are color-coded with similar colors representing high identity. Schemes are based on the TriTrypDB annotation (see http://tritrypdb.org). (PDF) [file pone.0168775.s004.pdf]
